# Diagnostic Dilemma: Which Clinical Tests Are Most Accurate for Diagnosing Supraspinatus Muscle Tears and Tendinosis When Compared to Magnetic Resonance Imaging?

**DOI:** 10.7759/cureus.25903

**Published:** 2022-06-13

**Authors:** Elif Balevi Batur, Pelin Zeynep Bekin Sarıkaya, Mustafa Emin Kaygısız, Ilknur Albayrak Gezer, Funda Levendoglu

**Affiliations:** 1 Physical Medicine and Rehabilitation, Selcuk University Faculty of Medicine, Konya, TUR; 2 Radiology, Kırıkkale University Faculty of Medicine, Kırıkkale, TUR

**Keywords:** diagnostic accuracy, impingement, shoulder pain, clinical shoulder tests, supraspinatus tear

## Abstract

Background: The study aims to investigate the diagnostic accuracy of five clinical shoulder tests in the diagnosis of supraspinatus tears and tendinosis when compared to magnetic resonance imaging (MRI).

Methods: A total of 116 shoulders of 106 consecutive patients who experienced shoulder pain were assessed for this cross-sectional diagnostic accuracy study. Patients were assessed with the most commonly used clinical shoulder tests, including the Jobe test (empty can), Neer test, drop arm test, Hawkins test, and full can test to identify supraspinatus tears and tendinosis. MRI examinations were performed on a 1.5 Tesla MRI system, and images were assessed by a blinded radiologist. The primary outcomes were to determine the sensitivity, specificity, and accuracy of the five clinical tests and to establish their correlation with MRI for supraspinatus tears and tendinosis.

Results: The Hawkins test had a higher sensitivity and accuracy when diagnosing tears (sensitivity 89.66% [95% CI, 78.83-96.11] and accuracy 56.03% [95% CI, 46.51-65.23], respectively) and higher sensitivity in tendinosis (79.07% [95% CI, 63.96-89.96]). The drop arm test had a lower sensitivity but higher specificity in both tendinosis and tears (sensitivity 0% [95% CI, 0-8.22] and 12.07% [95% CI, 4.99-23.29], respectively, and specificity 87.67% [95% CI, 77.88-94.21] and 96.5% [95% CI, 88.09-99.58], respectively). The Neer test had a higher positive predictive value (PPV) of 37.21% in diagnosing tendinosis. When compared to the Hawkins test, the combination of the clinical tests had no statistically significant contribution to sensitivity and diagnostic accuracy.

Conclusion: The Hawkins test had higher accuracy in diagnosing tears and was the most sensitive in diagnosing supraspinatus tendinosis and tears when compared to the MRI findings. The Neer test may also be another reliable tool for the diagnosis of tendinosis due to its higher PPV.

## Introduction

Shoulder pain is one of the most common musculoskeletal disorders. The incidence of shoulder disorders was predicted at 11.2/1000 patients/year, with most cases originating from rotator cuff tears [1-3], which are usually due to subacromial impingement syndrome (SIS). SIS is the most common pathology that causes shoulder pain, and the supraspinatus tendon is the most affected muscle by this syndrome [4-6]. In line with this, supraspinatus tendon tears constitute the most prevalent pathology that causes shoulder pain in approximately half of the patients presenting clinically. Tears also cause weakness and limited range of motion due to pain. A disruption in the tendon causes other rotator cuff muscles to become overloaded as they work as a force couple. Due to overloading by compensation, damage may occur in the intact tendons of other rotator cuff muscles. As a result, shoulder function may be wholly impaired [7].

The main clinical shoulder tests used to assay the continuity of the supraspinatus are the Jobe (empty can), full can, and drop arm tests [8]. However, the Neer and Hawkins tests, which are impingement tests, also play a role in the diagnosis of supraspinatus tears [9]. Patients with shoulder pain may be prediagnosed with these tests, and magnetic resonance imaging (MRI) maintains high sensitivity and specificity for diagnosing tear sizes and retraction [10].

Many studies in the literature have investigated the diagnostic accuracy of clinical shoulder tests; however, their results are contradictory [11,12]. In some studies, the results were reported individually only to patients undergoing surgery, which can lead to selection bias [13]. Furthermore, many of the studies were retrospectively designed, and the methodological content was inadequate [12]. Although MRI is the gold standard in diagnosis, it is well known that MRI could detect a high prevalence of rotator cuff tears in asymptomatic individuals [14]. These tears were most common in older adults and were consistent with the normal painless range of motion and functional activity [14,15]. Therefore, imaging modalities are not sufficient alone, and they should be combined with clinical shoulder tests to make precise diagnoses.

A recent study investigating the relationship between clinical tests and MRI and arthroscopy suggested that clinical tests might be a reliable tool for diagnosing shoulder pathologies [16]. To the best of our knowledge, there is little research investigating the combination of clinical shoulder tests in supraspinatus tendon tears and tendinosis in the literature. This study aimed to detect the supraspinatus pathologies with five different single tests and a combination of these tests to identify their accuracy for diagnosis when compared to MRI.

## Materials and methods

Participants were fully informed about the experimental procedures and gave their written informed consent. The inclusion criteria were patients aged 30-65 years with shoulder pain and limited range of motion for at least four weeks. Patients were excluded if they had a recent history of progressive degenerative changes, shoulder surgery of fractures, malignancy, adhesive capsulitis, rotator cuff pathologies other than supraspinatus, and infectious and inflammatory conditions of the shoulder. This study was consistent with the Standards for Reporting of Diagnostic Accuracy (STARD) guidelines and was approved by the ethical board committee of the university faculty of medicine (No: 2018.423).

Our clinical physical examination method was patterned after the original definitions of clinical shoulder tests defined in a meta-analysis by Gismervik et al. [17]. Tests were performed by an experienced single clinical physician in our physical medicine and rehabilitation department. The same physician performed all the examinations to prevent potential variability in the performance of physical examination maneuvers. The tests were considered positive if weakness and pain were detected on the affected shoulder.

Patients were assessed with the most commonly used clinical tests, including the Jobe (empty can) test, Neer test, drop arm test, Hawkins test, and full can test, to identify supraspinatus tears and tendinosis. In the Neer test, the patient was asked to sit; then, the physician put one hand on the patient’s scapula, and the other hand flexed the shoulder into the entire range. The test was considered positive if the pain occurred in the anterior or lateral shoulder with full flexion. The Jobe test (empty can) was performed with the arm at 90º abduction and 30º horizontal adduction and full internal rotation; then, the patient was asked to elevate the arm against resistance applied by the physician. Pain in the subacromial region of the shoulder and weakness indicated a positive test. When performing the full can test, the patient was asked to force the shoulder to elevate against resistance in 90° abduction, 30° horizontal adduction, and 45° external rotation. The test was positive if the pain was felt. The Hawkins test was performed by 90º shoulder flexion. The shoulder was then forced into internal rotation. Pain during this maneuver indicated a positive test. In the drop arm test, the patient’s shoulder was abducted passively at 90°; the patient was then asked to slowly lower the arm in the same arc. The drop arm test was considered positive if the patient could not controllably lower the arm. We also performed other clinical tests related to the infraspinatus, subscapularis, and biceps muscles. Of these, there were no positive results.

Magnetic resonance imaging (MRI) was used for this study as a reference standard test due to its high sensitivity and specificity. MRI examinations were performed on a 1.5 Tesla MRI system (Toshiba, Tokyo, Japan) with a dedicated shoulder coil. Each shoulder MRI examination contained a T2-weighted fat saturation sequence in the sagittal planes and proton-density sequences in the coronal and axial planes with a slice thickness of 3.5 mm and a gap of 0.5 mm. Images were analyzed by a blinded, trained musculoskeletal radiologist with 10 years of experience. Supraspinatus tendon images were divided into four classifications: Class 1: healthy patients without MRI findings, Class 2: supraspinatus tendinosis (increased T2-weighted MRI signals without tears), Class 3: supraspinatus partial tears (tears that did not extend from the articular surface to the bursal surface as well as intramuscular tears), and Class 4: supraspinatus total tears (tears that extended from the bursal surface to the articular surface).

Statistical analysis

The prevalence of shoulder impingement syndrome (the most common cause of shoulder pain) is estimated to be 30% in the literature. We calculated that a minimum sample size of 103 subjects was required to achieve a minimum power of 80% (actual power = 80.7%) for detecting a change in the percentage value of sensitivity of a diagnostic test from 0.70 to 0.90 based on a target significance level of 0.05 (actual p = 0.048) [2,18].

All statistical analyses were performed using R software v3.6.0 (www.r-project.org). The baseline values for the characteristics of the patients are presented as mean ± standard deviation, median (interquartile range) or counts (n), and percentage (%), as appropriate. The sensitivity, specificity, accuracy, predictive value, and likelihood ratio (LR) of the five clinical shoulder clinical tests were calculated using the 2 × 2 table method. These values were obtained with a 95% confidence interval (95% CI).

Moreover, dummy-coded sets of tests indicated whether one test, two tests, three tests, four tests, or all tests were positive. Combinations of tests were examined to improve the physicians’ ability to diagnose tears and tendinosis. The areas under the curve (AUC) of these combinations were also compared with the Hawkins test using receiver operating characteristic (ROC) curve analysis.

## Results

**Figure 1:**
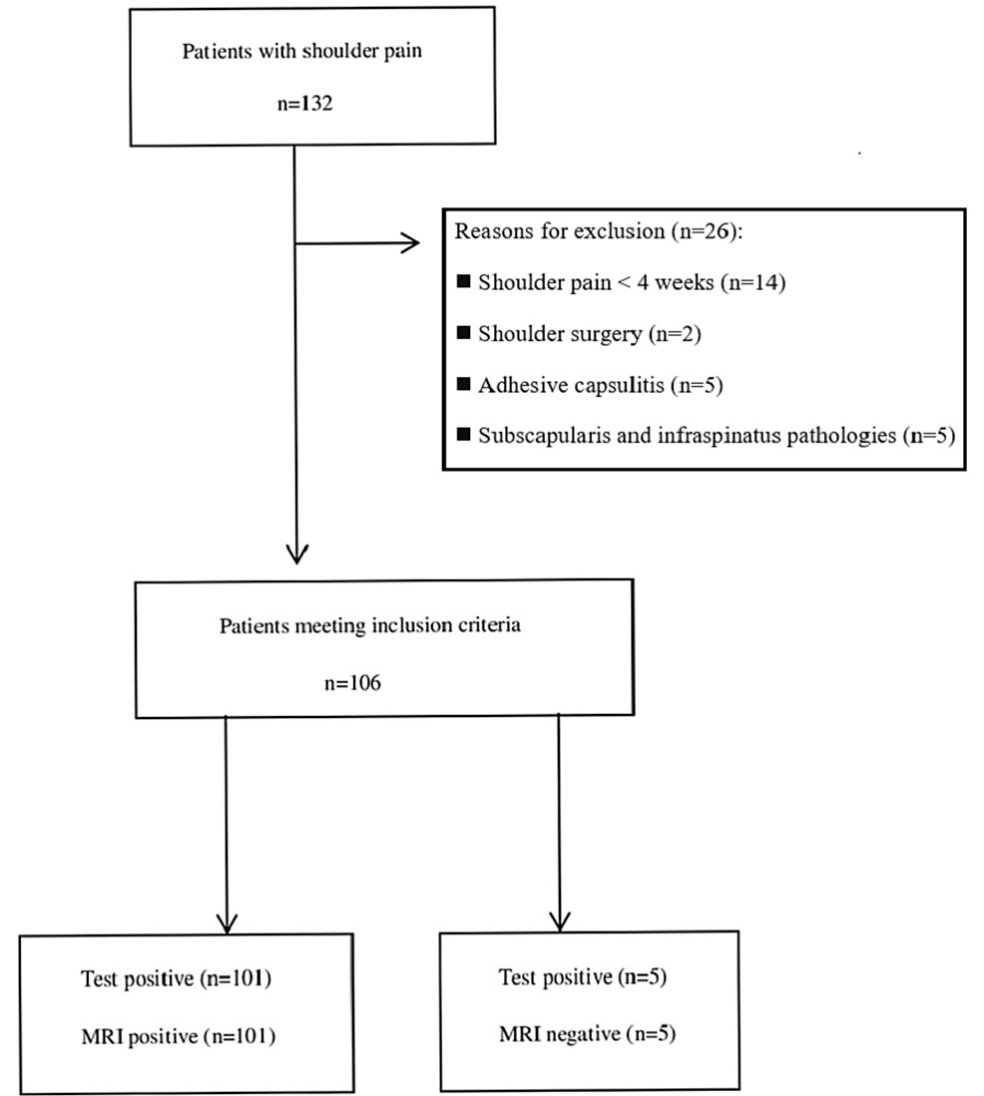
Flow chart of the study

In this cross-sectional diagnostic accuracy study, 132 individuals with shoulder pain were assessed for eligibility. Among them, 26 patients were excluded due to the following reasons: shoulder pain < four weeks (n = 14), shoulder surgery (n = 2), adhesive capsulitis (n = 5), subscapularis, and infraspinatus pathologies (n = 5). Finally, 106 patients were included in the study. The study flow chart is shown in Figure 1.

Patients did not receive any treatment for their shoulders until an MRI scan was performed. The mean age, height, weight, and body mass index (BMI) of the patients were 55.1 ± 10.2, 162.6 ± 10.1, 82.5 ± 14.4, and 31.3 ± 5.79, respectively; 32.08% of the patients were female. Further demographic characteristics of patients are shown in Table 1. In the full- and partial thickness tear group, the Hawkins test had a sensitivity of 89.66% and an accuracy of 56.03% in diagnosing tears. The drop arm test had a sensitivity of 12.07, a positive LR of 3.50 (0.75-16.14), a specificity of 96.55%, and a positive predictive value (PPV) of 77.78%. The positive and negative predictive values and 95% CI are shown in Table 2.

**Table 1:** Baseline characteristics of the patients Data are presented as mean ± standard deviation and median (interquartile range) or described as count (n) and percentages (%). BMI: Body mass index.

Characteristics	Patients
Age (years), mean ± SD	55.1 ± 10.2
Height (cm), mean ± SD	162.6 ± 10.1
Weight (kg), mean ± SD	82.5 ± 14.4
BMI (kg/m^2^), mean ± SD	31.3 ± 5.7
Gender, n (%)	
Male	34 (32.08%)
Female	72 (67.92%)
Side of the affected shoulder, n (%)	
Right	50 (47.17%)
Left	46 (43.40%)
Both	10 (9.43%)

**Table 2:** Diagnostic measures of the clinical shoulder tests with confidence intervals n^#^: Number of patients; TP: True positive; FN: False negative; FP: False positive; TN: True negative; LR: Likelihood ratio; PPV: Positive predictive value; NPV: Negative predictive value; MRI: Magnetic resonance imaging; CI: Confidence interval; NA: Not applicable; MRI+: Supraspinatus tear/tendinosis; *: Full and partial thickness tears are evaluated in the same group due to the small sample size of the full thickness.

Clinical tests		MRI+		MRI – Statistical diagnostic measures (95% CI)								
*Tears (full and partial thickness)	n^#^	Test + (TP)	Test – (FN)	Test + (FP)	Test – (FP)	Sensitivity	Specificity	Accuracy	LR+	LR–	PPV	NPV
Jobe test	58	44	14	38	20	75.86 (62.83 – 86.13)	34.48 (22.49 – 48.12)	55.17 (45.66 – 64.41)	1.16 (0.91 – 1.46)	0.70 (0.39 – 1.25)	53.66 (47.75 – 59.46)	58.82 (44.49 – 71.80)
Drop arm test	58	7	51	2	56	12.07 (4.99 – 23.29)	96.55 (88.09 – 99.58)	54.31 (44.81 – 63.59)	3.50 (0.75 – 16.14)	0.91 (0.82 – 1.01)	77.78 (43.14 – 94.17)	52.34 (49.66 – 55)
Full can test	58	44	14	40	18	75.86 (62.83 – 86.13)	31.03 (19.53 – 44.54)	53.45 (43.95 – 62.76)	1.10 (0.87 – 1.37)	0.78 (0.43 – 1.41)	52.38 (46.75 – 57.95)	56.25 (41.46 – 70)
Neer test	58	40	18	46	12	68.97 (55.45 – 80.46)	20.69 (11.17 – 33.35)	44.83 (35.58 – 54.34)	0.87 (0.70 – 1.08)	1.50 (0.80 – 2.83)	46.51 (41.17 – 51.93)	40 (26.14 – 55.67)
Hawkins test	58	52	6	45	13	89.66 (78.83 – 96.11)	22.41 (12.51 – 35.27)	56.03 (46.51 – 65.23)	1.16 (0.98 – 1.36)	0.46 (0.19 – 1.13)	53.61 (49.52 – 57.65)	68.42 (46.93 – 84.15)
Tendinosis	n^#^	Test + (TP)	Test – (FN)	Test + (FP)	Test – (FP)	Sensitivity	Specificity	Accuracy	LR+	LR-	PPV	NPV
Jobe test	43	28	15	54	19	65.12 (49.07 – 78.99)	26.03 (16.45 – 37.62)	40.52 (31.50 – 50.03)	0.88 (0.68 – 1.13)	1.34 (0.76 – 2.35)	34.15 (29.61 – 40.15)	55.88 (41.9 – 68.97)
Drop arm test	43	0	43	9	64	0 (0 – 8.22)	87.67 (77.88 – 94.21)	55.17 (45.66 – 64.41)	NA	1.14 (1.05 – 1.24)	NA	59.81 (57.73 – 61.86)
Full can test	43	31	12	53	20	72.09 (56.33 – 84.67)	27.39 (17.61 – 39.09)	43.97 (34.76 – 53.48)	0.99 (0.78 – 1.25)	1.02 (0.55 – 1.87)	36.90 (31.66 – 42.48)	62.50 (47.56 – 75.39)
Neer test	43	32	11	54	19	74.42 (58.82 – 86.48)	26.03 (16.45 – 37.62)	43.96 (34.76 – 53.48)	1.01 (0.81 – 1.25)	0.98 (0.52 – 1.86)	37.21 (32.19 – 42.52)	63.33 (47.67 – 76.61)
Hawkins test	43	34	9	63	10	79.07 (63.96 – 89.96)	13.69 (6.76 – 23.75)	37.93 (29.08 – 47.41)	0.92 (0.76 – 1.09)	1.53 (0.67 – 3.46)	35.05 (31.10 – 39.22)	52.63 (32.90 – 71.57)

In the tendinosis group, the Hawkins test had a sensitivity of 79.07% and an accuracy of 37.93% (29.08-47.41) in diagnosing tendinosis. The drop arm test had a higher specificity of 87.67%, an accuracy of 55.17%, and a sensitivity of 0% (0-8.22). The Neer test had a higher PPV of 37.21% in diagnosing tendinosis (Table 2). When a combination of the tests was performed in the case of at least one or two positive tests, the sensitivity for determining supraspinatus tears and tendinosis increased slightly. However, the PPV and LR+ values were found to have slightly decreased. There were no statistically significant differences between the Hawkins sign test and combinations of the tests (Table 3). There were no indeterminate results or missing responses in this study, as seen in Figure 1. Furthermore, there were no adverse events from performing shoulder clinical tests or the standard reference test (MRI).

**Table 3:** Comparison of the diagnostic measures between Hawkins test and combination of the single tests ROC: Receiver operating characteristic; AUC ± SE: Area under the curve ± standard error; LR: Likelihood ratio; PPV: Positive predictive value; NPV: Negative predictive value; MRI: Magnetic resonance imaging; CI: Confidence interval; NA: Not applicable.

Clinical tests	Comparison of ROC curves		Statistical diagnostic measures (95% CI)						
Tears* (Full and partial thickness)	AUC ± SE	p-value	Sensitivity	Specificity	Accuracy	LR+	LR–	PPV	NPV
Hawkins test	0.560 ± 0.034	[Reference]	89.66 (78.83 – 96.11)	22.41 (12.51 – 35.27)	56.03 (46.51 – 65.23)	1.16 (0.98 – 1.36)	0.46 (0.19 – 1.13)	53.61 (49.52 – 57.65)	68.42 (46.93 – 84.15)
At least 1 positive	0.509 ± 0.008	0.123	100 (93.8 – 100)	1.72 (0.04 – 9.2)	50.86 (41.42 – 60.26)	1.02 (0.98 – 1.05)	NA	50.43 (49.58 – 51.29)	100
At least 2 positive	0.509 ± 0.027	0.101	91.38 (81.02 – 97.14)	10.34 (3.89 – 21.17)	50.86 (41.42 – 60.26)	1.02 (0.91 – 1.15)	0.83 (0.27 – 2.58)	50.48 (47.53 – 53.42)	54.55 (27.94 – 78.78)
At least 3 positive	0.534 ± 0.041	0.521	75.86 (62.38 – 86.13)	31.03 (19.54 – 44.54)	53.45 (43.95 – 62.76)	1.10 (0.88 – 1.38)	0.78 (0.43 – 4.14)	52.38 (46.75 – 57.95)	56.25 (41.46 – 70)
At least 4 positive	0.552 ± 0.046	0.851	48.28 (34.95 – 61.78)	62.07 (48.37 – 74.49)	55.17 (45.66 – 64.41)	1.27 (0.83 – 1.94)	0.83 (0.61 – 1.15)	56 (45.45 – 66.03)	54.55 (46.57 – 62.30)
All 5 positive	0.534 ± 0.016	0.487	6.90 (1.91 – 16.73)	100 (93.84 – 100)	53.45 (43.95 – 62.76)	NA	0.93 (0.87 – 1)	100	51.79 (50.04 – 53.53)
Tendinosis	AUC ± SE	p-value	Sensitivity	Specificity	Accuracy	LR+	LR–	PPV	NPV
Hawkins test	0.536 ± 0.037	[Reference]	79.07 (63.96 – 89.96)	13.69 (6.76 – 23.75)	37.93 (29.08 – 47.41)	0.92 (0.76 – 1.09)	1.53 (0.67 – 3.46)	35.05 (31.10 – 39.22)	52.63 (32.90 – 71.57)
At least 1 positive	0.512 ± 0.011	0.498	97.67 (87.71 – 99.94)	0 (0 – 4.93)	36.21 (27.49 – 45.65)	0.98 (0.93 – 1.02)	NA	36.52 (35.46 – 37.60)	0
At least 2 positive	0.501 ± 0.028	0.538	90.70 (77.86 – 97.41)	9.59 (3.94 – 18.76)	39.66 (30.69 – 49.16)	1 (0.89 – 1.13)	0.97 (0.30 – 3.12)	37.14 (34.35 – 40.02)	63.64 (35.21 – 84.93)
At least 3 positive	0.540 ± 0.044	0.937	67.44 (51.46 – 80.92)	24.66 (15.32 – 36.14)	40.52 (31.50 – 50.03)	0.90 (0.70 – 1.14)	1.32 (0.73 – 2.38)	34.52 (29.20 – 40.27)	56.25 (41.66 – 69.83)
At least 4 positive	0.565 ± 0.047	0.542	34.88 (21.01 – 50.93)	52.05 (40.04 – 63.90)	45.69 (36.41 – 55.19)	0.73 (0.45 – 1.17)	1.25 (0.92 – 1.71)	30 (21.07 – 40.75)	57.58 (49.88 – 64.93)
All 5 positive	0.527 ± 0.013	0.822	0 (0 – 8.22)	94.52 (86.56 – 98.49)	59.48 (49.97 – 68.50)	0	1.06 (1 – 1.12)	0	61.61 (49.97 –68.50)

## Discussion

This study showed that the Hawkins test was the most sensitive test for supraspinatus tendinosis and tears. We also detected that the Hawkins test had the highest accuracy in diagnosing tears. Most of the prior studies reported that the Jobe test had a higher sensitivity and specificity; however, some studies suggested that the Hawkins test had a higher sensitivity, a result that aligns with our study [10,11,19]. 

In the present study, the Hawkins test was the most informative test, with a smaller LR negative ratio and good PPV compared to the other tests. On the other hand, compared to the Hawkins test, we found a high value of LR negative ratio in the Jobe test, indicating poor diagnostic accuracy. However, LR values should be examined with caution as in the drop arm test. We found that the drop arm test had a high specificity and low sensitivity that provided a greater LR positive value. The data may suggest that the positivity of the drop arm test indicated a supraspinatus tendon tear; indeed, due to its low sensitivity, it might not be suitable for use as a screening test. Our findings regarding this test were in line with Jain et al.’s cohort study [20]. In another study, Mac-Donald et al. compared the Hawkins test and the Neer test in 85 patients who underwent diagnostic arthroscopy. Consistent with our study, they found that the Hawkins test had a higher sensitivity than the Neer test (88% and 85%, respectively) [21]. Regarding shoulder biomechanics and mechanical characteristics, it was declared in a study that the supraspinatus tendon was mostly compressed with abduction and internal rotation positions [22]. Therefore, Hawkins tests might be superior in diagnosing supraspinatus pathologies.

We evaluated our results with tests that assess the supraspinatus tendon based on MRI. To prevent confusion in the results, we did not allow the participation of patients with other rotator cuff pathologies. In addition, only one physician performed the tests to avoid variability in clinical evaluations. Our study determined that the Neer test had a higher PPV for diagnosing tendinosis. This result was similar to a study by Fodor et al. that found that the Neer test had a high PPV in SIS. In contrast to our research, they used ultrasonographic images when comparing the accuracy of the clinical shoulder tests [23].

In our study, we also assessed the accuracy of the combination of single tests for diagnosing supraspinatus tears and tendinosis. As a result, when compared to the Hawkins test, no statistically significant contribution to sensitivity or diagnostic accuracy was detected. In line with these results, Somerville et al. suggested that there was no optimal combination of tests that improves sensitivity for the diagnosis of rotator cuff tears [24]. Considering these findings, performing the tests in combination may not be useful for the diagnosis. A clinical diagnostic test should differentiate between healthy people and patients [22]. In diagnosing tears, we detected that the drop arm test had high specificity and PPV but low sensitivity. These data are similar to the study of Jain et al., which suggested that a negative drop arm test does not exclude underlying supraspinatus tears or tendinosis [20]. Therefore, using the drop arm test may not be suitable for diagnosis. 

In this study, MRI was preferred for evaluating the supraspinatus tendon due to the high resolution of scans and the accuracy of showing retraction and tear sizes. However, it must be noted that rotator cuff tears may be detected on MRI in asymptomatic individuals and may significantly increase with older age [6,14]. In light of this information, we would like to emphasize the necessity of evaluating patients with clinical shoulder tests before using MRI.

Study limitations

In the present study, we did not evaluate the relationship between clinical tests and partial and full-thickness tears due to the small number of patients with full-thickness tears. We evaluated both of them in the same group. This is one of the limitations of the study. Another limitation is that we did not analyze the length of tears and their relationship with clinical tests. Further studies with a larger sample size are needed to obtain more generalizable results.

## Conclusions

Our results show that the Hawkins test had a higher sensitivity in supraspinatus tendinosis and tears when correlated with MRI. Also, in diagnosing tears, the Hawkins test had a higher accuracy than other tests. Therefore, the Hawkins test may be considered one of the most reliable tests for diagnosing SIS as the supraspinatus tendon is mainly affected by this syndrome. For tendinosis, the Neer test may also be considered a reliable tool due to its higher PPV. As a result, combining imaging techniques with clinical tests may provide precise results in diagnosis.
